# Template-Sacrificing Synthesis of Ni-Co Layered Double Hydroxides Polyhedron as Advanced Anode for Lithium Ions Battery

**DOI:** 10.3389/fchem.2020.581653

**Published:** 2020-12-08

**Authors:** Youjun Lu, Yingjie Du, Haibo Li

**Affiliations:** ^1^School of Materials Science and Engineering, North Minzu University, Yinchuan, China; ^2^Ningxia Key Laboratory of Photovoltaic Materials, Ningxia University, Yinchuan, China

**Keywords:** energy storage, layered double hydroxides, polyhedron, electrochemistry, anode

## Abstract

The novel hollowed Ni-Co layered double hydroxide polyhedron (H-(Ni, Co)-LDHP) is synthesized via a template-sacrificing approach using ZIF-67 as template. The morphology, crystallinity, porous texture, and chemical state of H-(Ni, Co)-LDHP are examined. It demonstrates that the H-(Ni, Co)-LDHP not only provides rich redox sites but also promotes the kinetics due to presence of numerous rational channels. As a result, the H-(Ni, Co)-LDHP manifests the desirable lithium ions storage performance when employed as anode. This study paves a new way for preparing hollowed nanostructure toward advanced electrochemical applications.

## Introduction

Currently, the environmental tolerance is priority to the development of new energy storage devices which are of great significance to our daily life (He et al., 2018). Among all candidates, lithium ion batteries (LIBs) are considered to be the suitable one due to many advantages such as high energy density, high specific capacity, and long lifetime (Li et al., 2019).

Layered double hydroxides (LDHs) are hydrotalcite-like compounds. The general formula of LDH can be expressed as [${\text{M}}_{1-\text{x}}^{2+}$M^3+^_*x*_(OH)_2_] [A^n−^]_x/*n*_·*z*H_2_O (Chen et al., 2013) where M^2+^ generally indicates the Mg^2+^, Zn^2+^, Ni^2+^, and Cu^2+^, and M^3+^ represents the Al^3+^, Ga^3+^, Fe^3+^, and Mn^3+^. Moreover, the LDH often exhibits the laminated structure which is composed of exchangeable interlayer anions and positively charged host layers (Wang and O'Hare, 2012). In the past few years, the LDHs have been widely applied in many fields, such as catalysis, photoelectricity, and bioengineering (Zand et al., 2019). Owing to the presence of abundant active sites and layered structure, the expected redox reaction incorporated short diffusion lengths of ions and electrons are affiliated with LDH, making it a potential anode for LIBs. However, the poor conductivity and serious aggregation of LDH nanosheets restricts its lithium storage performance (Xuan et al., 2019). Based on many cases, one of the effective strategies is to modify LDHs with rational carbon materials coatings enabling the improved conductivity and expected electrochemical properties, such as aerogel, graphene nanosheets, and carbon nanotubes (Gao et al., 2011; Yang et al., 2013; Song et al., 2017).

Metal organic frameworks (MOFs) representing a class of unique 3D carbon materials are synthesized via linking metal ions with organic molecules. As a result, the MOFs have superior advantages of well-defined porosity, controllable particle size, and tunable electronic structure. Most significantly, MOF have been extensively proposed and developed as sacrificing template for the synthesis of LDH^@^C nanocomposites for electrochemical applications since it is able to derive the desirable transition metal oxides and sufficient specific surface area (Furukawa et al., 2013; Gao et al., 2019).

In this work, we developed a facile strategy to prepare hollowed Ni-Co layered double hydroxide polyhedron [H-(Ni, Co)-LDHP]. The typical ZIF-67 was synthesized initially and then employed as sacrificing template and Co source to couple with nickel ions, forming polyhedron-like Ni-Co LDH with hollowed nanostructure. The hollowed structures have shown significant advantages of huge specific surface area and excellent mass permeability enabling to create plenty of active sites and promote charge transfer during the charge/discharge process and further enhance the electrochemical performance. The structure, crystallinity, porous texture, and chemical state are examined using transmission electron microscopy (TEM), high-resolution TEM (HRTEM), X-ray diffraction (XRD), nitrogen adsorption-desorption, and X-ray photoelectron spectrum (XPS). Moreover, the electrochemical performances of H-(Ni, Co)-LDHP have been explored by applying H-(Ni, Co)-LDHP as anode for LIBs.

## Experimental

### Preparation of H-(Ni, Co)-LDHP

Firstly, 30 mmol of Co(NO_3_)_2_6H_2_O and 120 mmol of dimethylimidazole were dissolved in 400 ml mixed solution consisting of methanol and absolute ethanol in a volume ratio of 1:1. After stirring for 5 min, the mixed solution was placed for 24 h, and then the purple precipitates which are ZIF-67 nanoparticles were collected. Subsequently, 120 mg of ZIF-67 powders and 400 mg Ni(NO_3_)_2_6H_2_O were dissolved in 100 ml absolute ethanol, which was then subjected to ultrasonic bath for 90 min. The ultrasonic power was 120 W. Finally, the green H-(Ni, Co)-LDHP powders were obtained via centrifugation and washed three times using absolute ethanol.

### Electrochemical Tests

The cyclic voltammetry (CV) curves and electrochemical impedance spectroscopy (EIS) within the frequency range from 0.1 to 10 000 Hz were carried out on electrochemical workstation (Princeton PARSTAT 3000-DX, USA). The galvanostatic charging/discharging curves (GCD) were measured on Land battery test system (CT2001A, China). All tests were performed under a CR2032 coins-type cell which was assembled in a glove box filled with argon gas. Regarding the cell, the lithium metal foil and Celgard membrane were employed as counter electrode and separator, respectively. The active materials, acetylene carbon black and polyvinylidene fluoride (PVDF), were dissolved in N-methylpyrrolidinone (NMP) in a mass ratio of 8:1:1 to fabricate electrode, and the average mass loading of anode is 10 mg. Besides, 1 M LiPF_6_ in ethylene carbonate (EC), propylene carbonate (PC), and Dimethyl Carbonate (DEC) (1:1:1 V/V/V) was employed as the electrolyte. In general, the volume of electrolyte is set as 1.2 ml.

## Results and Discussion

**Figure 1:**
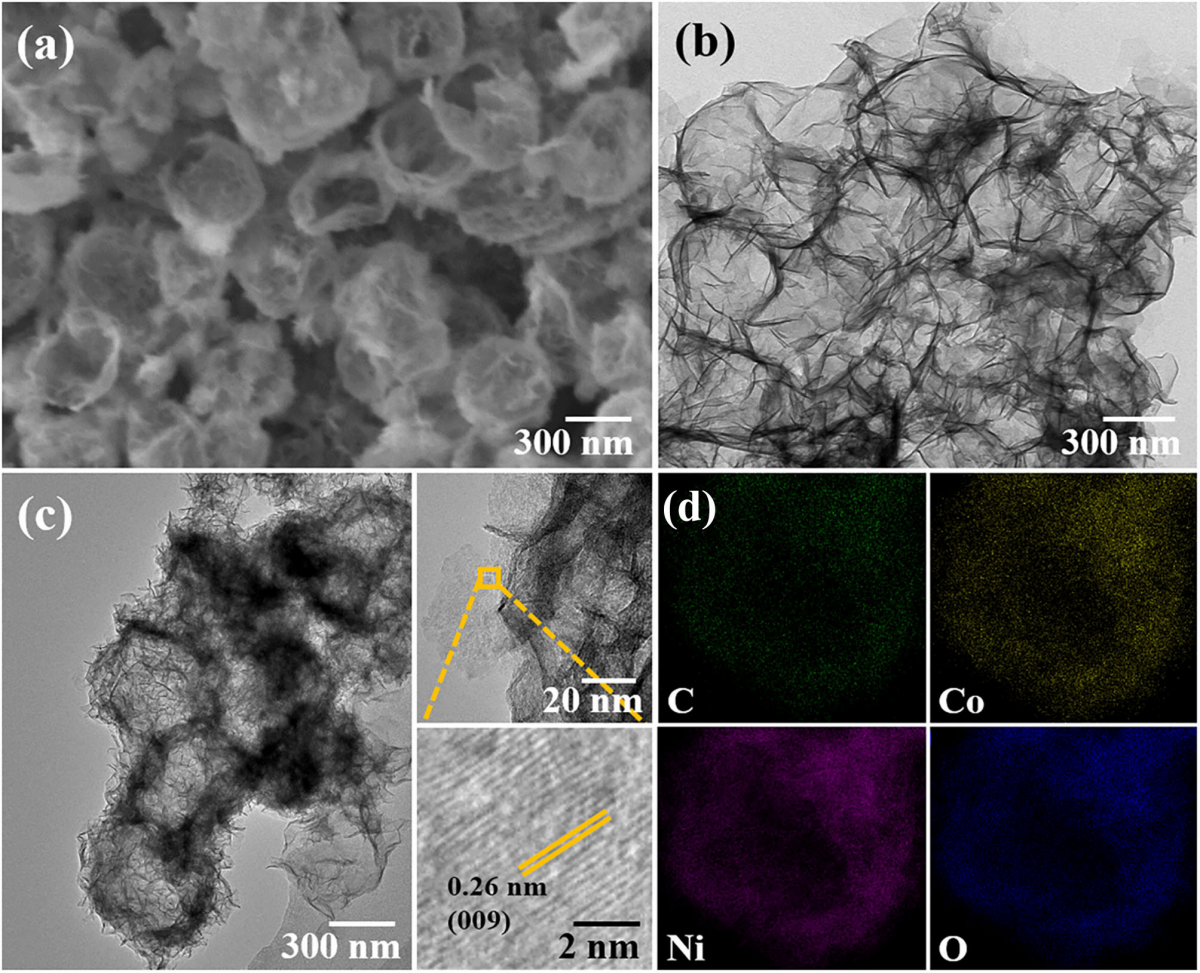
SEM **(a)** and TEM **(b)** image of H-(Ni, Co)-LDHP, **(c)** HRTEM images, and **(d)** element mapping image of H-(Ni, Co)-LDHP.

Figures 1a,b show the scanning electron microscope (SEM, Hitachi SU5000, Japan) and transmission electron microscopy (TEM, Hitachi HT7700, Japan) images of H-(Ni, Co)-LDHP, respectively. Through employing ZIF-67 as sacrificing template, the irregular Ni-Co LDH hollow polyhedral nanocages were obtained. Roughly, the average geometrical size of H-(Ni, Co)-LDHP is 500 nm. Beyond that, the fine structure of H-(Ni, Co)-LDHP is explored by high-resolution TEM (HRTEM, FEI TalosF200s, USA). In Figure 1c, the H-(Ni, Co)-LDHP is assembled by a large number of nanosheets. It is proposed that the multistage structure is beneficial to increase the specific surface area (SSA) and expose as much as active cites for the diffusion of electrolyte ions, which promote the reaction kinetics and thus contribute to high LIBs' performance. Further, the distinct lattice fringe spacing of 0.26 nm corresponding to the (009) crystal plane of NiCo-LDHs is verified from the inset of Figure 1c, indicating the good crystallinity of LDHs. Figure 1d is the combined elemental mapping image of H-(Ni, Co)-LDHP, suggesting the even distribution of C, Co, Ni, and O. According to the respective atomic fraction of C, Co, and Ni, the content of carbon is thereby obtained as 3.45%. Further, the element mapping reveals that LDH nanosheets uniformly disperse on ZIF-67, suggesting that the ZIF-67 as sacrificing template effectively inhibits the agglomeration of LDH and improves the specific surface area. The mechanism of MOF-derived hollow structure can be briefly stated as follows: ion exchange initiates the interchange of cation/anions during the synthesis of MOF-directed hollowed structures. When excess Ni(NO_3_)_2_ and ZIF-67 are dissolved in absolute ethanol, the hydrolysis of Ni^2+^ ions would etch the surface of ZIF-67. Meanwhile, Co^2+^ released from ZIF-67 is partially oxidized by O_2_ and NO^3−^ ions (Hu et al., 2015; Xu et al., 2018b). Afterwards, the Co^2+^ and Ni^2+^ consume large amount of hydroxide ions to co-precipitate around the ZIF-67 polyhedron forming an LDH layer. Besides, the continuous ultrasonic vibration and outflow of Co^2+^ ions empty ZIF-67 polyhedron, leading to the formation of hollowed Ni-Co layered double hydroxide polyhedron.

**Figure 2:**
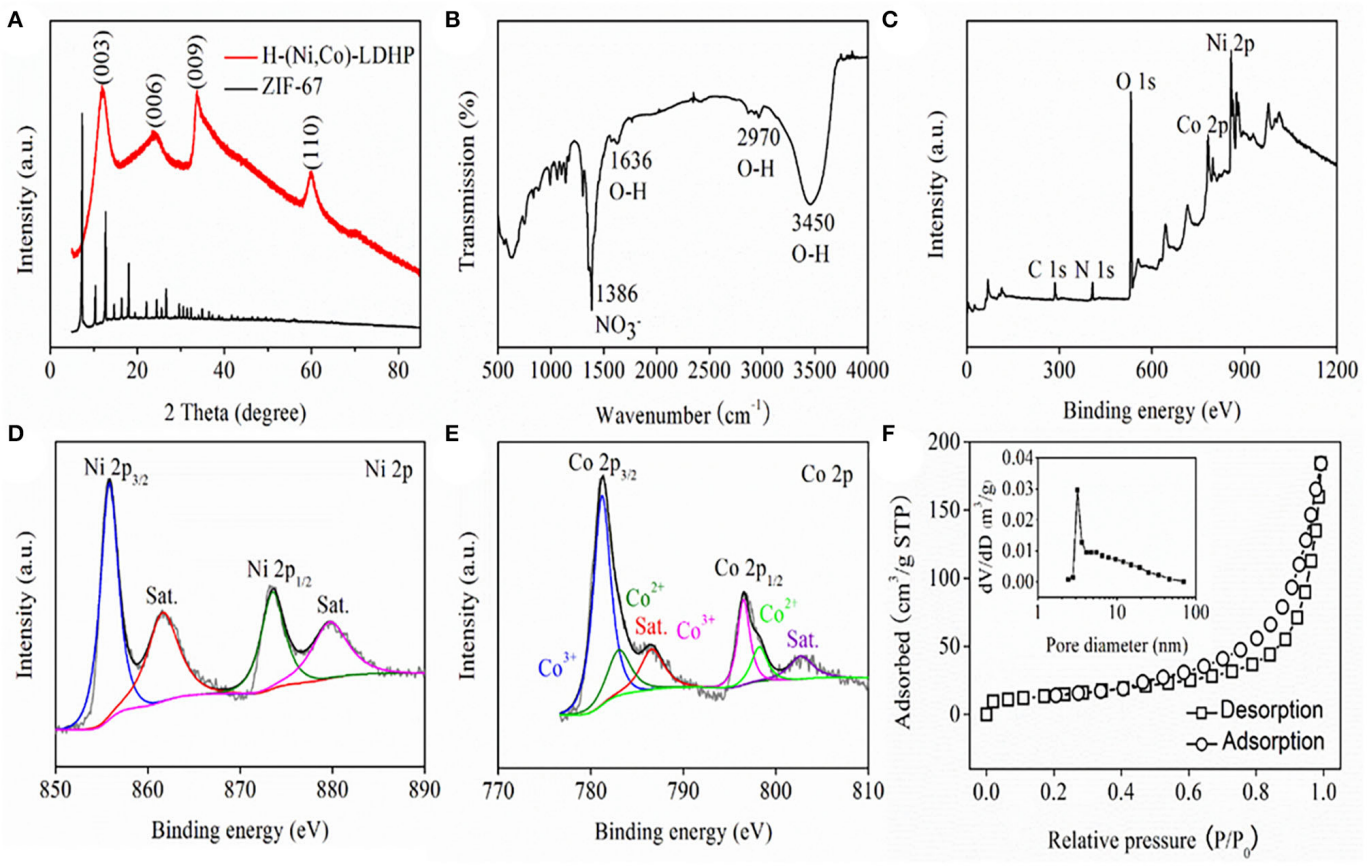
**(A)** XRD pattern, **(B)** FTIR spectrum, **(C)** XPS survey spectrum, **(D)** high-resolution Ni 2p spectrum, **(E)** high-resolution Co 2p spectrum, and **(F)** Nitrogen adsorption-desorption isotherm and pore size distribution curve of H-(Ni, Co)-LDHP.

Figure 2A exhibits the X-ray diffraction (XRD, Rigaku Smart Lab, Japan) pattern of H-(Ni, Co)-LDHP and ZIF-67 precursor. The XRD pattern of ZIF-67 corresponds to previous report very well (Kong et al., 2018). Regarding H-(Ni, Co)-LDHP, the peaks located at 11.6, 23.8, 33.7, and 60.4° are ascribed to (003), (006), (009), and (110) crystal plane of hydrotalcite-like NiCo-LDH (Jiang et al., 2013). Figure 2B illustrates the Fourier transform infrared spectroscopy spectrum (FTIR PerkinElmer S2, USA) of H-(Ni, Co)-LDHP. The broad band emerging at 3,450 cm^−1^ is associated to O-H stretching mode of hydrogen-bonded hydroxyl, and the unimpressive bands presenting at 1,636 and 2,970 m^−1^ are assigned to the water molecules in the interlayer and adsorbed on the sample (Hu et al., 2019). Further, the intense band focusing at 1,386 cm^−1^ is ascribed to the N-O vibration mode of NO^3−^ (Xu et al., 2018a). Besides, the successive band in the low wavenumber ranging from 500 to 1,000 cm^−1^ are identified as the stretching vibrations of M-O-H (M represents Ni or Co) (Tang et al., 2015). The component elements and chemical states of H-(Ni, Co)-LDHP were detected by X-ray photoelectron spectrum (XPS, Thermo Fischer DXR, USA). The full XPS survey spectrum shown in Figure 2C verifies the existence of C, N, O, Co, and Ni. Figure 2D draws the Ni 2p spectrum of H-(Ni, Co)-LDHP. Two dominant peaks showing at 855.8 and 873.5 eV with spin-energy separation of 17.7 eV indicate the presence of Ni^2+^, together with two satellite peaks located at 861 and 879.7 eV, respectively (Lee et al., 2018). In terms of the high-resolution spectra of Co 2p (Figure 2E), two peaks emerging at 781.2 and 796.5 eV are resulted from Co^3+^, while the binding energy corresponds to 783 and 798.2 eV conforming the appearance of Co^2+^ (Su et al., 2019). The pore texture and SSA of sample were investigated by nitrogen adsorption-desorption system (JW-BK200, CHN). As implied in Figure 2F, it manifests distinct hysteresis loop which signifies the imbalance of adsorption-desorption process due to the hollow structure of H-(Ni, Co)-LDHP. Note that the SSA of H-(Ni, Co)-LDHP is 60.65 m^2^/g which is superior to traditional NiCo-LDHs nanosheets in previous reports, originating from the unique nanostructure (Qian et al., 2019). Meanwhile, the pore size distribution curve extracted from the inset of Figure 2F demonstrates that the dominant pore size is 3 nm, which is favorable for penetrating Li^+^, resulting in high kinetics.

**Figure 3:**
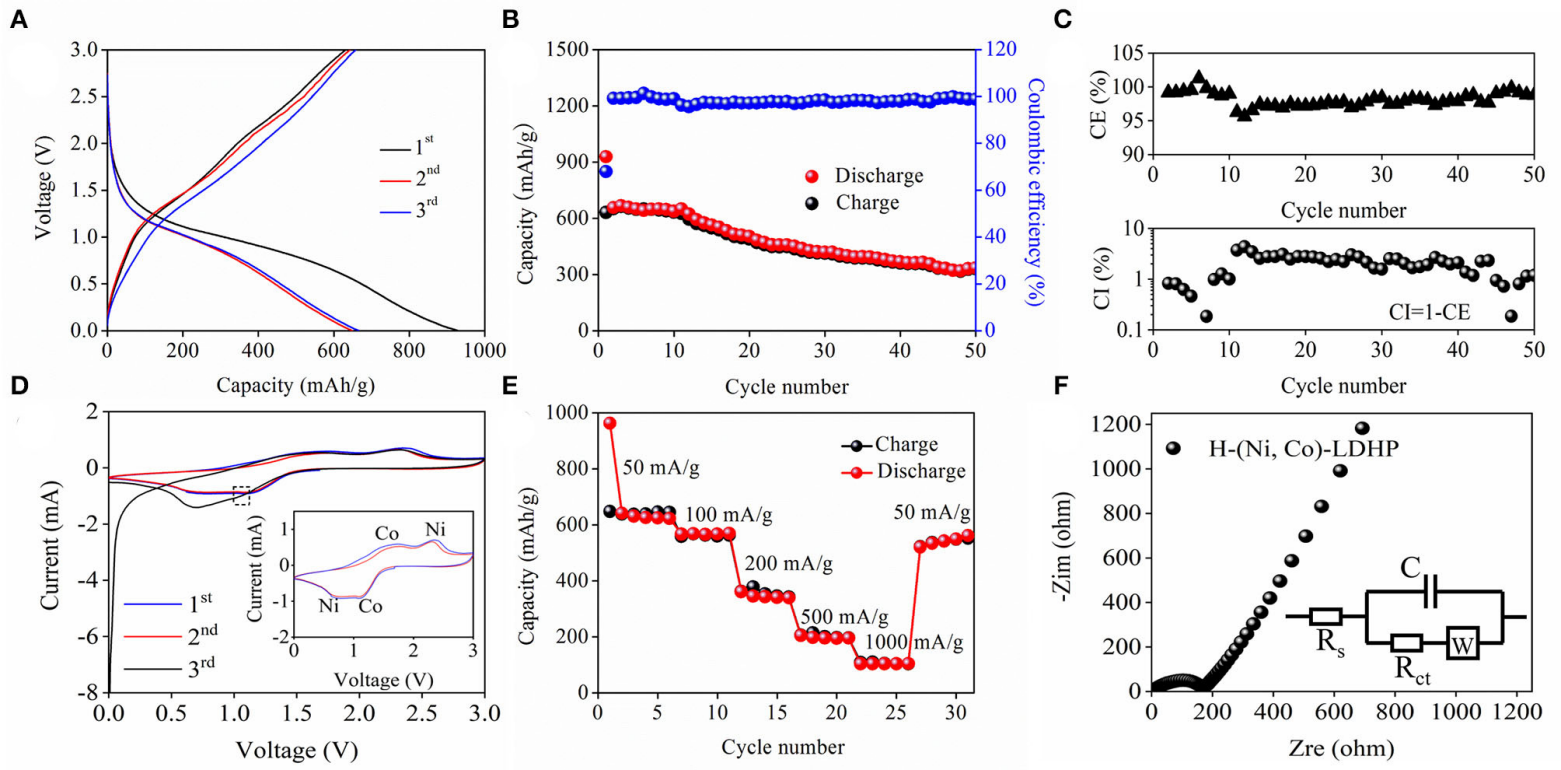
**(A)** GCD curves of H-(Ni, Co)-LDHP at current density of 100 mA/g, **(B)** cycling performance and Columbic efficiency, **(C)** enlarged Columbic efficiency curve, **(D)** CV curves, **(E)** rate capability, and **(F)** Nyquist plots of the H-(Ni, Co)-LDHP.

Figure 3A shows the first, second, and third GCD profiles of H-(Ni, Co)-LDHP anode at current density of 100 mA/g. In the first circle, the initial discharge capacity is 928.3 mAh/g, and the reversible charge capacity is 630.8 mAh/g, illustrating that the coulumbic efficiency (CE) is 68%. The inferior CE can be attributed to the formation of steady solid electrolyte interphase (SEI) film. The obvious discharge plateau appears from 0.6 to 1.2 V which is in accordance with the characteristic peaks from CV curves. The cyclic performance and CE are exhibited in Figure 3B. As it is shown, the discharge capacity decreased from 928.3 to 335.4 mAh/g after 50 cycles at current density of 100 mA/g, suggesting 36.1% capacitance retention. In terms of the CE, the initial value increased from 68 to 98% after subsequent cycle and then became stable. Besides the SEI film, the unexpected CE should be ascribed to the crack of H-(Ni, Co)-LDHP associated with the porous hollowed microstructure and low carbon content. Furthermore, there is obvious downtrend in CE after undergoing several times cycles in Figure 3C. Actually, the frequently insertion and de-insertion of Li^+^ damages the nanoarchitecture of H-(Ni, Co)-LDHP, leading to serious collapse. As a result, the fresh SEI is generated on the surface of cracked H-(Ni, Co)-LDHP, which continuously consumes electrolyte and transferable Li^+^, causing the fact that the CE only maintains at 98%. On the other hand, the coulombic inefficiency (CI) is equal to 1-CE, which is helpful to evaluate the lithiation reversibility of H-(Ni, Co)-LDHP. In Figure 3C, the fluctuant CI curve again verifies the formation of unstable SEI layer and continuous capacity attenuation of H-(Ni, Co)-LDHP anode. Figure 3D draws the CV curves of H-(Ni, Co)-LDHP anode at scan rate of 0.1 mV/s with the potential range from 0 to 3 V. In the first curve, a deflected anodic peak at 1.03 V is assigned to the formation of SEI film. In the subsequent curves, the cathodic peaks at 0.63 V can be ascribed to conversion of Ni^2+^ to Ni^0^, and anodic peak at 2.32 V is attributed to oxidation of Ni^0^ to Ni^2+^. Meanwhile, the reduction peak at 1.13 V and oxidation peak at 1.73 V represent the redox reactions of ionic Co^2+^ and Co^0^. Figure 3E exhibits the rate capability of H-(Ni, Co)-LDHP anode. When the current density is set as 50, 100, 200, 500, and 1,000 mA/g, the discharge capacity stabilizes at 641.6, 566, 361, 204.8, and 103.6 mAh/g, respectively. Significantly, it is found that the specific capacity reached 520.4 mAh/g when the current density returned to 1,000 mA/g from 50 mA/g. Figure 3F demonstrate the Nyquist plots of H-(Ni, Co)-LDHP, and equivalent circuit (insert). In simulation, the *R*_s_ and *R*_ct_ represent the resistance of Li^+^ passing through the SEI and charge transfer process, respectively. The Warburg resistance (W) is on behalf of the diffusion rate of Li^+^ in the electrolyte. Originating from the fitting, the *R*_s_ and *R*_ct_ can be calculated, which are 5.66 and 170.7 Ω, respectively. Such low value indicates the superior reaction kinetics and preferable electron transport characteristics.

## Conclusion

In summary, the H-(Ni, Co)-LDHP has been synthesized by a rational template-sacrificing approach. Benefiting from the abundant active sites and convenient diffusion path of charge transfer, the H-(Ni, Co)-LDHP anode exhibited delivered high specific capacity (928.3 mAh/g at 100 mA/g). Thus, this work provides a new method to design hollow LDH nanocages for high specific capacity LIBs.

## Data Availability Statement

The original contributions generated for the study are included in the article/supplementary material, further inquiries can be directed to the corresponding author/s.

## Author Contributions

YL, HL, and YD analyzed the data and wrote the manuscript. All authors contributed to the article and approved the submitted version.

## Conflict of Interest

The authors declare that the research was conducted in the absence of any commercial or financial relationships that could be construed as a potential conflict of interest.
